# Liver metastases in advanced urothelial carcinoma (ARON-2): do pembrolizumab and avelumab make a difference in a poor-prognosis scenario?

**DOI:** 10.3389/fimmu.2026.1667155

**Published:** 2026-02-24

**Authors:** Linda Cerbone, Giandomenico Roviello, Fabio Calabrò, Tarek Taha, Enrique Grande, Kirstin Binz, Ravindran Kanesvaran, Alina Pirstuk, Ondřej Fiala, Javier Molina-Cerrillo, Teresa Alonso-Gordoa, Alessandro Rizzo, Renate Pichler, Zin W. Myint, Alexandr Poprach, Gaetano Facchini, Melichar Bohuslav, Hana Studentova, Franco Morelli, Tomas Buchler, Alessia Mennitto, Deniz Tural, Luigi Formisano, Inmaculada Orejana, Martina Catalano, Sebastiano Buti, Fernando Sabino Marques Monteiro, Andrey Soares, Shilpa Gupta, Francesco Massari, Matteo Santoni

**Affiliations:** 1 Istituto di Ricovero e Cura a Carattere Scientifico (IRCCS), National Cancer Institute Regina Elena, Rome, Italy; 2 Department of Health Sciences, Section of Clinical Pharmacology and Oncology, University of Florence, Florence, Italy; 3 The Royal Marsden National Health Service (NHS) Foundation Trust, The Institute of Cancer Research, London, United Kingdom; 4 Department of Medical Oncology, MD Anderson Cancer Center Madrid, Madrid, Spain; 5 Division of Medical Oncology, Department of Internal Medicine, University of Kansas Cancer Center, Kansas City, KS, United States; 6 Division of Medical Oncology, National Cancer Centre Singapore, Singapore, Singapore; 7 Department of Oncology, Second Faculty of Medicine, Charles University and University Hospital Motol, Prague, Czechia; 8 Department of Oncology and Radiotherapeutics, Faculty of Medicine and University Hospital in Pilsen, Charles University, Pilsen, Czechia; 9 Biomedical Center, Faculty of Medicine in Pilsen, Charles University, Pilsen, Czechia; 10 Department of Medical Oncology, Hospital Ramón y Cajal, Madrid, Spain; 11 S.S.D. C.O.r.O. Bed Management Presa in Carico, TDM, Istituto di Ricovero e Cura a Carattere Scientifico (IRCCS) Istituto Tumori “Giovanni Paolo II,”, Bari, Italy; 12 Department of Urology, Comprehensive Cancer Center Innsbruck, Medical University of Innsbruck, Innsbruck, Austria; 13 Division of Medical Oncology, Department of Internal Medicine, Markey Cancer Center, University of Kentucky, Lexington, KY, United States; 14 Masaryk Memorial Cancer Institute, Masaryk University, Brno, Czechia; 15 Oncology Operative Unit, Santa Maria delle Grazie Hospital, Azienda Sanitaria Locale (ASL) NA2 NORD, P, ozzuoli, Napoli, Italy; 16 Department of Oncology, Faculty of Medicine and Dentistry, Palacký University, Olomouc, Czechia; 17 Medical Oncology Unit, Istituto di Ricovero e Cura a Carattere Scientifico (IRCCS) Casa Sollievo della Sofferenza, Foggia, Italy; 18 Department of Oncology, Charles University and University Hospital Motol, Prague, Czechia; 19 Department of Medical Oncology, Azienda Ospedaliera Universitaria “Maggiore Della Carità,”, Novara, Italy; 20 Department of Medical Oncology, Koc University Medical Faculty, Istanbul, Türkiye; 21 Department of Medicine and Surgery, Federico II University, Naples, Italy; 22 Medical Oncology Unit, University Hospital of Parma, Parma, Italy; 23 Department of Medicine and Surgery, University of Parma, Parma, Italy; 24 Oncology and Hematology Department, Hospital Sírio Libanês, Brasília, Brazil; 25 Latin American Cooperative Oncology Group (LACOG), Porto Alegre, Brazil; 26 Oncology Unit, Hospital Israelita Albert Einstein, São Paulo, Brazil; 27 Taussig Cancer Institute, Cleveland Clinic, Cleveland, OH, United States; 28 Medical Oncology, Istituto di Ricovero e Cura a Carattere Scientifico (IRCCS) Azienda Ospedaliero-Universitaria di Bologna, Bologna, Italy; 29 Department of Medical and Surgical Sciences (DIMEC), University of Bologna, Bologna, Italy; 30 Medical Oncology Unit, Macerata Hospital, Macerata, Italy

**Keywords:** avelumab, immunotherapy, liver metastases, NCT05290038, pembrolizumab, urothelial carcinoma

## Abstract

**Background:**

Over the past decade, the treatment landscape for metastatic urothelial carcinoma (mUC) has improved significantly with the introduction of immunotherapy, targeted agents, and antibody–drug conjugates. The median overall survival (mOS) reached 36.7 months in cisplatin-eligible and 25.6 months in cisplatin-ineligible patients in the first-line setting and over 10 months post-platinum failure. However, liver metastases remain a known poor prognostic factor.

**Methods:**

We conducted a retrospective analysis of mUC patients treated at 79 global institutions. Two cohorts were defined: cohort 1 included patients who progressed after platinum-based therapy and received pembrolizumab, and cohort 2 included patients who received avelumab as maintenance therapy. Treatments were administered between 1 January 2016 and 31 October 2024.

**Results:**

Cohort 1 (n = 1,341) had an mOS of 17.5 months. Patients without liver metastases had significantly longer OS than those with liver involvement (20.1 *vs*. 9.4 months, p <0.001). Among patients with liver metastases, OS was 11.8 months in males *vs*. 5.8 months in females (p = 0.066). OS was longer in those with BMI ≥25 kg/m² (14.1 *vs*. 8.1 months, p = 0.028) and better ECOG-PS (ECOG 0: 17.0 months; ECOG 1: 9.8; ECOG ≥2: 3.1; p <0.001). Cohort 2 (n = 291) had an mOS of 25.8 months. Again, OS was longer in patients without liver metastases (27.0 *vs*. 16.4 months, p <0.001). Among those with liver involvement, OS was 14.7 months in males and 20.0 months in females (p = 0.310). Patients with BMI ≥25 had non-reached OS versus 17.1 months in those with lower BMI (p <0.001). ECOG-PS remained a strong prognostic factor (NR for ECOG 0; 14.7 months for ECOG 1; 4.6 months for ECOG ≥2, p <0.001).

**Conclusion:**

Liver metastases are associated with significantly reduced survival in patients with mUC receiving immunotherapy. However, both pembrolizumab and avelumab demonstrated improved outcomes compared with historical chemotherapy data. These findings underscore the need for personalized treatment strategies in high-risk subgroups.

## Introduction

Bladder cancer, with approximately 614,000 new cases and 220,000 deaths in 2022, is the ninth most common tumor worldwide. In recent years, mortality rates have declined in some countries, probably because of reductions in smoking and improvements in treatments (1). Survival rates are strictly related to the stage at diagnosis, with a 5-year relative survival rate ranging from 97% for early stages to 7%–9% for metastatic disease (2). In developed countries, urothelial carcinoma (UC) is metastatic at presentation in 5% of cases, while 50% of patients (pts) have muscle-invasive bladder cancer relapsing after curative treatment (3). In less developed regions (LMIC), the percentage of upfront metastatic disease rises to 40% (4). For decades, the overall survival (OS) of patients with metastatic UC has been affected by eligibility to receive cisplatin-based chemotherapy, with a median OS (mOS) of approximately 14 months in platinum-eligible and 9 months in platinum-ineligible patients (5–8). After platinum failure, mOS with subsequent chemotherapy regimens such as taxanes or vinflunine has been reported to be between 5 and 7 months (9–11). Nevertheless, in the last 10 years, with the advent of immunotherapy, targeted agents, and antibody–drug conjugates, the mUC treatment landscape has evolved significantly, with mOS in the first-line setting reaching 36.7 months in cisplatin-eligible patients, and 25.6 months in cisplatin-ineligible patients, and mOS after platinum failure setting reaching more than 10 months (12–15). Different prognostic factors have been investigated in mUC, and the presence of liver metastases has been shown in many studies to be associated with poor outcomes (16, 17). Generally, the liver is the third most common site of metastasis for mUC patients (after lymph nodes and lung), with a prevalence in the first- or later-line setting ranging from 17% to 26%, respectively (16, 18). Many authors have discussed the apparent lack of efficacy of immunotherapy in this specific setting of patients; however, are we sure that new drugs are ineffective with the presence of liver metastases, or is the apparent lack of efficacy related to other negative prognostic factors? (19). A recent comparative real-world analysis examined the outcomes of pembrolizumab and avelumab in mUC (20). In this study, no significant differences in OS or PFS were observed between patients receiving pembrolizumab after platinum failure and those receiving avelumab maintenance has been reported. These findings reinforce the need to understand prognostic modifiers, such as liver metastases, which may influence treatment choice and outcomes across settings. Our study, a sub-analysis of the ARON-2 study (21), expands this evidence by specifically evaluating the prognostic impact of liver metastases in two clinically distinct cohorts.

## Methods

### Study design and population

**Figure 1:**
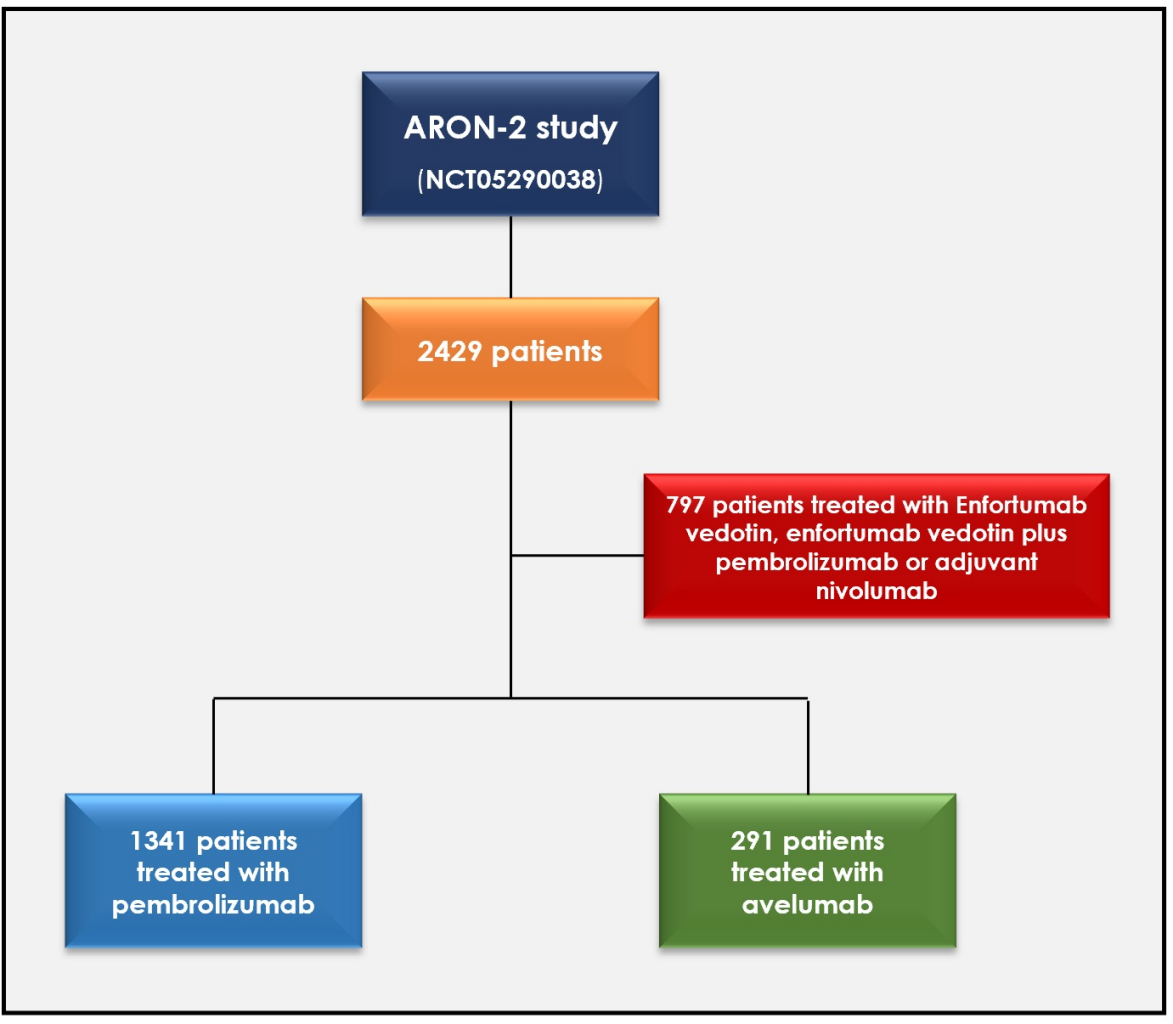
Flowchart of the study design.

We conducted a retrospective analysis of the clinical data of patients with UC aged ≥18 years with radiologically confirmed metastatic disease. Two cohorts were defined in this study. Cohort 1 included patients with either progressive or recurrent disease after platinum-based treatments who were subsequently treated with pembrolizumab, whereas in cohort 2, patients received avelumab as maintenance therapy after first-line platinum-based chemotherapy and achieved at least stable disease (**Figure 1**). Patients were treated with one of these two drugs between 1 January 2016 and 31 October 2024, and data were collected from 79 institutions worldwide.

All participants had available data on demographic factors (age and sex), tumor histology, Eastern Cooperative Oncology Group-Performance Status (ECOG-PS), metastatic sites, prior surgeries, chemotherapy treatments, treatment durations, and response to pembrolizumab or avelumab, as assessed by the Response Evaluation Criteria in Solid Tumors (RECIST) version 1.1. Body Mass Index (BMI) was calculated as weight in kilograms divided by the square of height in meters.

Data on clinical and pathological characteristics were collected from the medical and pathology records of each center, as per routine clinical practice. Standard physical examinations, laboratory tests, and imaging studies (computed tomography and magnetic resonance imaging scans) were performed according to local guidelines.

Patients with incomplete clinical or outcome data were excluded.

### Ethical considerations

The study protocol was approved by the Ethical Committee of the coordinating center (Marche Region, Italy; approval number 2022 39, Study Protocol “ARON 2 Project”) and by the Institutional Review Boards of the participating centers. This study adhered to the Good Clinical Practice (GCP), the Declaration of Helsinki, and international ethical standards for biomedical research. Informed consent was obtained from all patients, while consent was waived for patients who died or were lost to follow-up by the Institutional Review Board of the coordinating center.

### Study objectives

The primary aim of this study was to evaluate the prognostic role of liver metastases in patients with metastatic UC treated with pembrolizumab (cohort 1) or avelumab (cohort 2). Tumor responses (progression disease [PD], stable disease [SD], partial response [PR], and complete response [CR]) were evaluated based on the RECIST version 1.1 criteria. We collected data on the overall response rate (ORR) and OS. OS was defined as the time from pembrolizumab or avelumab initiation until death from any cause.

### Statistical analysis

To compare OS across groups, we used the Kaplan–Meier method and the log-rank test. The median follow-up time was determined using the Kaplan–Meier method. Cox proportional hazard models were employed to evaluate the multivariable impact on patient survival and estimate hazard ratios (HRs) with 95% confidence intervals (CIs). Fisher’s exact test was used for pairwise comparisons of categorical variables, and chi-square tests were used for multiple categorical comparisons. Statistical significance was set at p <0.05.

To mitigate confounding factors associated with the retrospective, non-randomized study design, a propensity score matching (PSM) approach was applied. Propensity scores were derived from a multivariable logistic regression model incorporating age, sex, ECOG performance status, tumor histology, primary tumor location, site of metastases, and type of immunotherapy. Patients receiving avelumab were matched in a 1:1 ratio with those treated with pembrolizumab using nearest-neighbor matching without replacement, applying a caliper of 0.2 standard deviations of the logit-transformed propensity score. Post-matching covariate balance was evaluated using standardized mean differences (SMDs), with thresholds below 0.1 indicating an adequate balance. The primary and secondary outcomes of the study were subsequently reassessed in the matched population using Kaplan–Meier survival analyses and Cox proportional hazards regression models, as appropriate.

## Results

### Overall study population

We included 1,632 patients from the ARON-2 dataset; 1,064 (65%) were male and 568 (35%) were female. Two hundred and twenty (13%) patients had an ECOG-PS ≥2. Pure urothelial carcinoma histology was reported in 79% of patients, and 1,184 (73%) reported tumors of the lower urinary tract.

Cohort 1 included 1,341patients treated with pembrolizumab, and cohort 2 included 291 patients treated with avelumab. Patient’ characteristics are summarized in **Table 1**.

**Table 1:** Patient characteristics. Cohort 1 (pembrolizumab) and cohort 2 (avelumab).

Characteristics	Overall study population	*Pembrolizumab (cohort 1)*				*Avelumab (cohort 2)*			
		Overall no. (%)	Patients with liver metastases no. (%)	Patients without liver metastases no. (%)	*P-value*	Overall no. (%)	Patients with liver metastases no. (%)	Patients without liver metastases no. (%)	*P-value*
Total patients	1,632 (100)	1,341 (100)	234 (100)	1,107 (100)	–	291 (100)	51 (100)	240 (100)	–
Sex Male Female	1,064 (65) 568 (35)	835 (62) 506 (38)	137 (59) 97 (41)	698 (63) 409 (47)	0.664	229 (79) 62 (21)	39 (76) 12 (24)	190 (79) 50 (21)	0.735
Median age (years)	71	70	69	71	–	73	71	73	–
Current or former smokers Yes No	1,029 (63) 603 (37)	835 (62) 506 (38)	137 (59) 97 (41)	698 (63) 409 (47)	0.664	194 (67) 97 (33)	30 (59) 21 (41)	164 (68) 76 (32)	0.240
BMI ≤25 Kg/m^2^ >25 Kg/m^2^	937 (57) 695 (43)	767 (57) 574 (43)	136 (58) 98 (42)	631 (57) 476 (43)	1.000	170 (58) 121 (42)	35 (68) 16 (32)	135 (56) 105 (44)	0.109
ECOG performance status 0 1 ≥2	516 (32) 896 (55) 220 (13)	423 (32) 724 (54) 194 (14)	64 (27) 124 (53) 46 (20)	359 (32) 600 (54) 148 (14)	0.474	93 (32) 172 (59) 26 (9)	11 (22) 30 (58) 10 (20)	82 (34) 142 (59) 16 (7)	**0.012**
Tumor histology Pure urothelial carcinoma Minor or mixed variants	1,296 (79) 336 (21)	1,045 (78) 296 (22)	181 (77) 53 (23)	864 (78) 243 (22)	1.000	251 (86) 40 (14)	43 (84) 8 (16)	208 (87) 32 (13)	0.689
Primary tumor location Upper urinary tract Lower urinary tract	448 (27) 1,184 (73)	364 (27) 977 (73)	74 (32) 160 (68)	290 (26) 817 (74)	0.436	84 (29) 207 (71)	12 (24) 39 (76)	72 (30) 168 (70)	0.345
Metastatic disease Synchronous Metachronous	534 (33) 1,098 (67)	425 (32) 916 (68)	92 (39) 142 (61)	333 (30) 774 (70)	0.234	109 (37) 182 (63)	26 (51) 25 (49)	83 (35) 157 (65)	**0.032**
Common sites of metastasis Lymph nodes (non-regional) Lung Bone Brain	1,068 (65) 563 (34) 441 (27) 24 (1)	864 (64) 458 (34) 373 (29) 23 (2)	152 (65) 105 (45) 74 (32) 9 (4)	712 (64) 353 (32) 299 (27) 14 (1)	1.000 0.081 0.444 0.214	204 (70) 105 (36) 68 (23) 1 (1)	35 (69) 16 (31) 15 (29) 1 (1)	169 (70) 89 (37) 53 (22) 0 (0)	1.000 0.456 0.330 1.000

In bold: Statistically significant value (p<0.05).

### Pembrolizumab (cohort 1)

#### Patient population

Cohort 1 included 1,341 patients treated with pembrolizumab for progressive or recurrent disease after platinum-based treatments from the ARON-2 dataset; 835 (62%) were men and 506 (38%) were women. The median follow-up time was 22.9 months (95%CI: 19.6–24.7). One hundred ninety-four patients (14%) had an ECOG-PS ≥2. Pure UC histology was reported in 78% of patients; 977 (73%) and 364 (27%) patients reported tumors of the bladder and upper urinary tract, respectively. Synchronous metastatic disease was reported in 425 (32%) patients.

Two hundred and thirty-four patients (17%) treated with pembrolizumab presented with liver metastases.

The most frequent concomitant metastatic sites were distant lymph nodes (65%) and the lungs (45%). Patient characteristics are summarized in **Table 1**.

#### Outcome analysis in the overall study population treated with pembrolizumab

**Figure 2:**
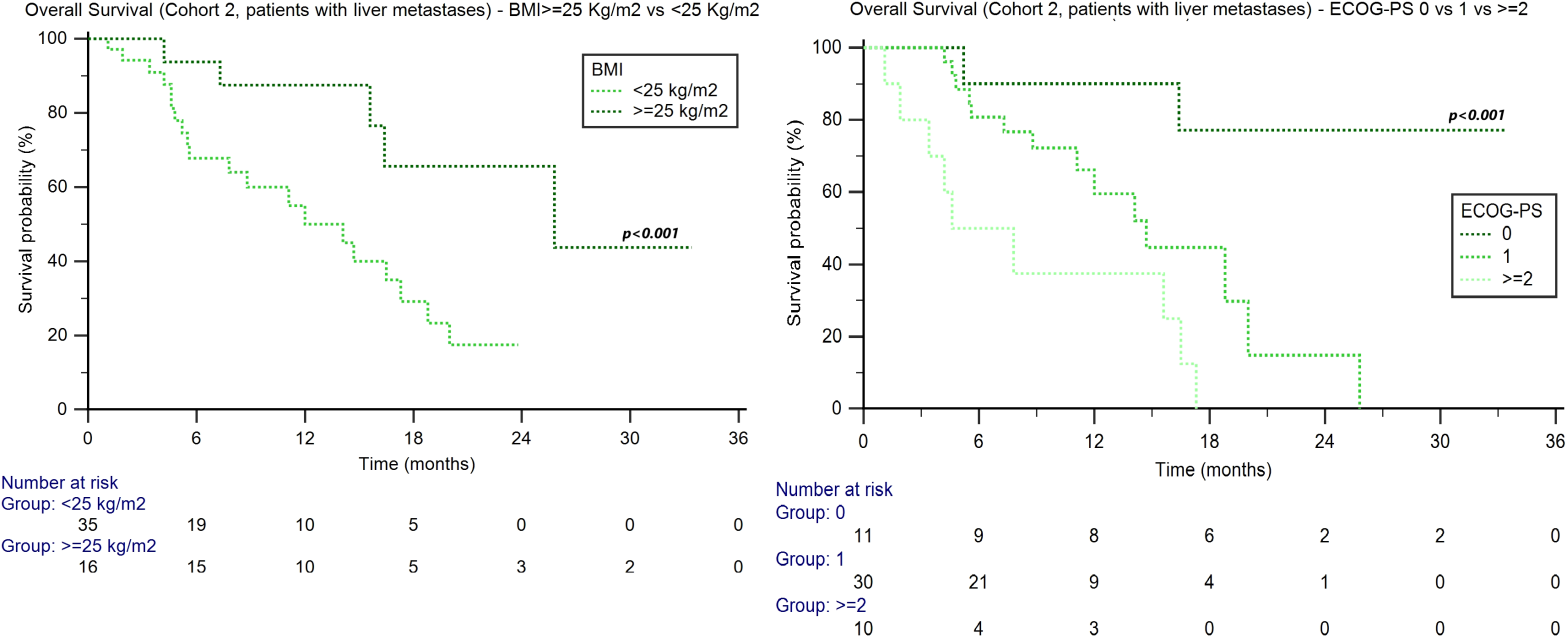
Overall survival in advanced UC patients with or without liver metastases treated with pembrolizumab (cohort 1) or avelumab (cohort 2).

The median OS was 17.5 months (95%CI: 15.9–88.9) in cohort 1 and was significantly longer in patients without liver metastases (20.1 months, 95%CI: 17.9–88.9 *vs*. 9.4 months, 95%CI: 7.4–72.4, *p* < 0.001, **Figure 2**). In cohort 1, 709 patients (53%) had died at the time of analysis, whereas the rate was higher among patients with liver metastases (156 patients, 67%; *p* = 0.045). Of the 576 patients who progressed during pembrolizumab treatment, 201 (34.8%) went on to receive a further line of treatment.

#### Outcome analysis in patients with liver metastases treated with pembrolizumab

**Figure 3:**
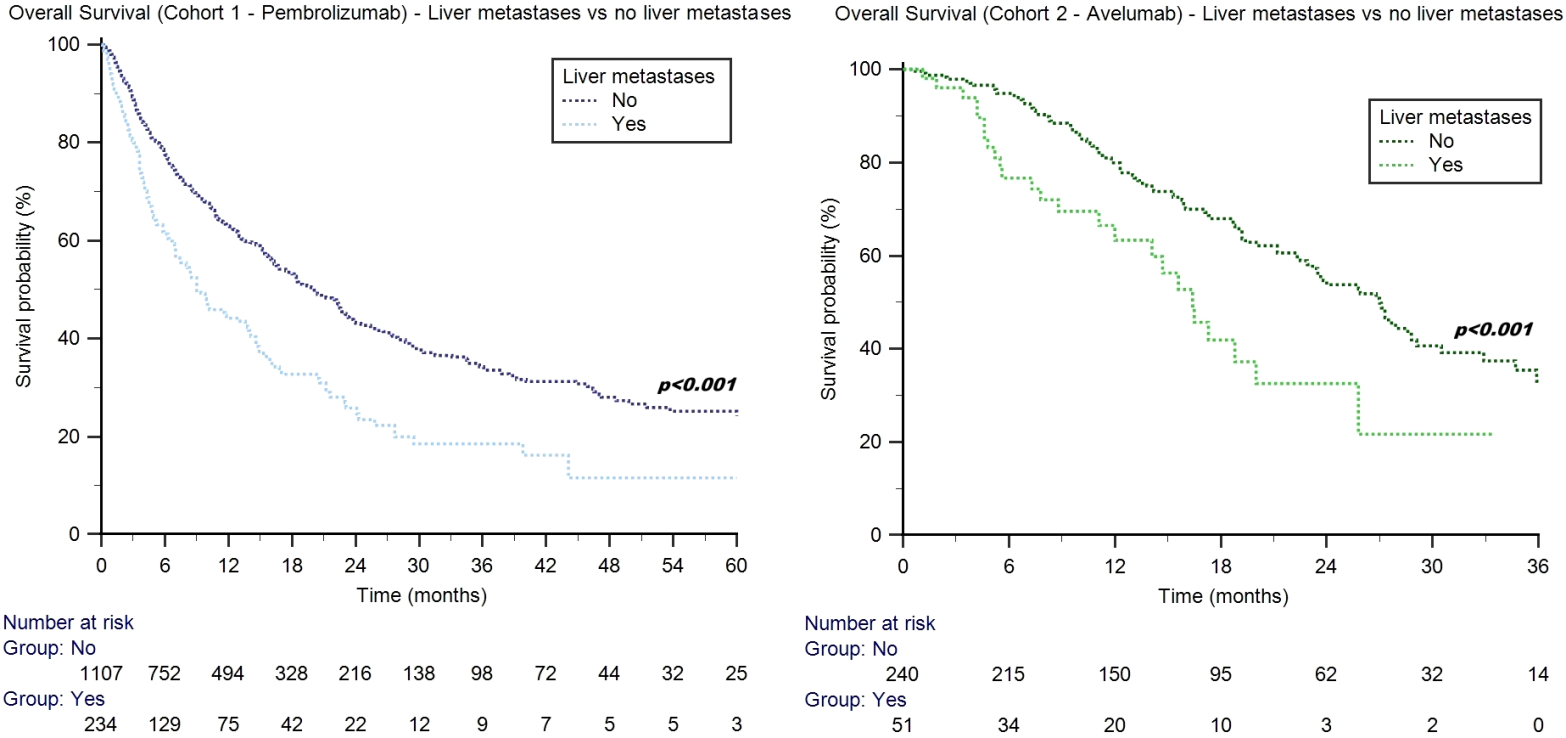
Overall survival in advanced UC patients with liver metastases treated with pembrolizumab (cohort 1) stratified by BMI, ECOG-PS, *de novo* metastatic disease, and bone metastases.

In patients with liver metastases, the median OS was 11.8 months (95%CI: 9.0–72.4) in males and 5.8 months (95%CI: 4.3–8.3) in females (*p* = 0.066). No significant differences were found between patients aged ≥70 y and those aged <70 y (8.0 months, 95%CI: 4.8–39.8 *vs*. 10.2 months, 95%CI: 8.4–72.4; *p* = 0.497). The median OS was 14.1 months (95%CI: 9.0–21.2) in patients with a BMI ≥25 kg/m^2^ and 8.1 months (95%CI: 5.2–72.4) in subjects with a BMI <25 kg/m^2^ (*p* = 0.028, **Figure 3**).

The median OS was 17.0 months (95%CI: 13.9–72.4) in patients with ECOG-PS = 0, 9.8 months (95%CI: 8.1–13.8) in subjects with ECOG-PS = 1, and 3.1 months (95%CI: 1.8–23.1) in the subgroup with ECOG-PS ≥2 (*p* < 0.001, **Figure 3**).

No statistically significant differences were found between current or former smokers and non-smokers (9.8 months, 95%CI: 8.4–72.4 *vs*. 7.4 months, 95%CI: 5.2–14.1; *p* = 0.539).

We further stratified the patients according to tumor histology and primary site. No statistically significant differences of median OS were found between patients with pure and mixed urothelial histology (9.8 months, 95%CI: 7.0–14.6 *vs*. 8.4 months, 95%CI: 5.8–72.4; *p* = 0.333) or between patients with lower and upper tract UC (10.2 months, 95%CI: 7.0–14.6 *vs*. 8.0 months, 95%CI: 4.8–72.4; *p* = 0.818).

The median OS was longer in patients with metachronous metastatic disease (14.0 months, 95%CI: 8.4–16.4 *vs*. 6.7 months, 95%CI: 4.0–72.4; *p* = 0.008, **Figure 3**).

When we stratified patients according to the site of metastases, the median OS was shorter in patients with liver plus bone metastases (5.2 months, 95%CI: 3.8–9.0 *vs*. 11.7 months, 95%CI: 8.4–72.4; *p* = 0.032, **Figure 3**), while no statistically significant differences were found in patients with additional metastases to distant lymph nodes (9.9 months, 95%CI: 7.0–44.1 *vs*. 9.0 months, 95%CI: 5.9–72.4; *p* = 0.989) and lungs (8.1 months, 95%CI: 5.8–13.1 *vs*. 9.8 months, 95%CI: 8.0–72.4; *p* = 0.325). The trend of shorter survival in patients with brain metastases did not reach statistical significance (median 4.1 months, 95%CI: 1.1–6.9 *vs*. 9.8 months, 95%CI: 8.0–72.4; *p* = 0.124), possibly due to the limited size of this subgroup.

#### Response to pembrolizumab

In cohort 1, we observed 8% CR, 22% PR, 24% SD, and 46% PD (ORR = 30%). In patients with liver metastases, we observed 7% CR, 16% PR, 16% SD, and 61% PD (ORR = 23%). No statistically significant differences were found between the ORR in patients with or without liver metastases, while the rate of patients presenting with PD as the best response to pembrolizumab was significantly higher in patients with liver metastases (*p* = 0.047).

#### Univariable and multivariable analyses

We first performed univariable and multivariable analyses in cohorts 1 and 2 together, showing that BMI, ECOG-PS, synchronous metastatic disease, and bone, liver, and brain metastases were significantly associated with OS (**Supplementary Table S1**).

We further performed univariable and multivariable analyses in cohort 1. In the univariate analysis, BMI, ECOG-PS, synchronous metastatic disease, and lung, bone, liver, and brain metastases were significantly associated with OS. In the multivariable analysis, only lung metastases did not retain prognostic significance (**Supplementary Table S2**).

Furthermore, we performed additional univariable and multivariable sub-analyses of patients from cohort 1 with liver metastases, showing that BMI, ECOG-PS, synchronous metastatic disease, and bone metastases were significantly associated with OS (**Supplementary Table S3**).

### Avelumab (Cohort 2)

#### Patient population

Cohort 2 included 291 patients from the ARON-2 dataset treated with avelumab; 229 (79%) were men. The median follow-up time was 21.8 months (95%CI: 18.0−66.1). Two hundred and sixty-five patients (91%) had an ECOG-PS of 0–1. Pure UC histology was reported in 251 patients (86%), with 207 patients (71%) presenting with tumors of the lower urinary tract. Synchronous metastatic disease was reported in 109 (37%) patients. Fifty-one patients (18%) had liver metastases. Concomitant metastases to the lymph nodes or lungs were reported in 70% and 36% of patients, respectively. The characteristics of patients receiving avelumab maintenance therapy are summarized in **Table 1**.

### Outcome analysis in patients receiving avelumab

The median OS was 25.8 months (95%CI: 21.2–27.5) in cohort 2 and was significantly longer in patients without liver metastases (27.0 months, 95%CI: 23.2–29.1 *vs*. 16.4 months, 95%CI: 12.0–20.0; *p* < 0.001, **Figure 2**). In cohort 2, 122 patients (42%) were dead at the time of analysis, and the rates were 49% and 40% in patients with and without liver metastases, respectively (*p* = 0.255). A total of 204 patients (85%) out of 239 who progressed during avelumab treatment received further lines of treatment.

#### Outcome analysis in patients with liver metastases treated with avelumab

In cohort 2, patients with liver metastases showed a median OS of 14.7 months (95%CI: 7.8–18.8) in males and 20.0 months (95%CI: 12.0–25.8) in females (*p* = 0.310). No significant differences were found between patients aged ≥70 y *vs*. <70 y (27.0 months, 95%CI: 22.9–29.1 *vs*. 21.2 months, 95%CI: 17.5–27.3, *p* = 0.643). The median OS was NR in patients with a BMI ≥25 kg/m^2^ and 17.1 months (95%CI: 14.1–20.0) in subjects with a BMI <25 kg/m^2^ (HR: 0.30, 95%CI: 0.21–0.43, *p* < 0.001, **Figure 3**).

**Figure 4:**
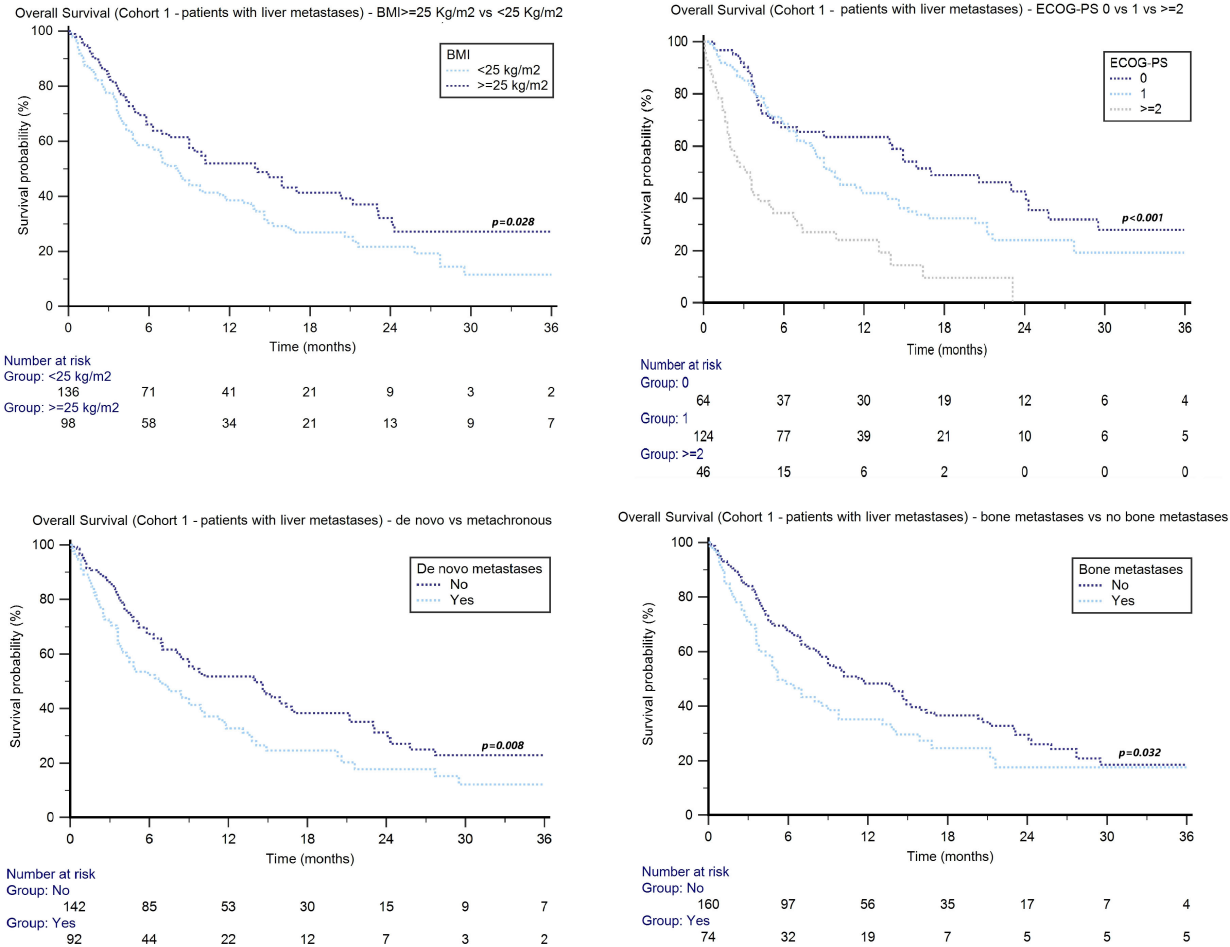
Overall survival in advanced UC patients with liver metastases treated with avelumab (cohort 2) stratified by BMI and ECOG-PS.

The median OS was NR in patients with ECOG-PS = 0, 14.7 months (95%CI: 11.1–25.8) in subjects with ECOG-PS = 1, and 4.6 months (95%CI: 1.1–17.3) in the subgroup with ECOG-PS ≥2 (*p* < 0.001, **Figure 4**).

No statistically significant differences were found between current or former smokers and non-smokers (14.7 months, 95%CI: 5.5–18.8 *vs*. 20.0 months, 95%CI: 14.1–22.2; *p* = 0.073).

We further stratified patients according to tumor histology, primary site, and time to metastatic disease. No statistically significant differences in terms of median OS were found between patients with pure and mixed urothelial histology (16.4 months, 95%CI: 11.1–25.8 *vs*. 18.8 months, 95%CI: 1.9–20.8; *p* = 0.796), patients with lower *vs*. upper tract UC (15.6 months, 95%CI: 8.8–18.8 *vs*. not reached, NR, *p* = 0.058). or patients with metachronous *vs*. synchronous metastatic disease (18.8 months, 95%CI: 7.3–25.8 *vs*. 14.7 months, 95%CI: 7.8–16.5; *p* = 0.441).

When we stratified patients according to the site of metastases, the median OS was not significantly different in patients with or without distant lymph node (14.7 months, 95%CI: 8.8–25.8 *vs*. 16.5 months, 95%CI: 7.8–17.3; *p* = 0.651), bone (16.5 months, 95%CI: 4.8–20.0 *vs*. 16.4 months, 95%CI: 11.1–25.8; *p* = 0.624), or lung metastases (14.1 months, 95%CI: 5.2–25.8 *vs*. 16.5 months, 95%CI: 7.8–20.0; *p* = 0.713).

#### Response to avelumab

In cohort 2, the response consisted of 11% CR, 15% PR, 39% SD, and 35% PD (ORR = 26%). In patients with liver metastases, we observed 10% CR, 17% PR, 33% SD, and 40% PD (ORR = 27%). No statistically significant differences were found between the ORR (*p* = 1.000) and the rate of patients presenting with PD as the best response to avelumab (*p* = 0.559).

#### Univariate and multivariate analyses

We performed univariate and multivariate analyses in cohort 2. BMI, ECOG-PS, and liver metastases were significantly associated with OS in both univariable and multivariable analyses (**Supplementary Table S4**).

Furthermore, we performed additional univariable and multivariable analyses in patients with liver metastases from cohort 2, showing that BMI and ECOG-PS were significantly associated with OS in univariate analyses, while only ECOG-PS confirmed its prognostic role in multivariate analysis (**Supplementary Table S5**).

## Discussion

Tumor metastasis is a hallmark of advanced-stage cancers and profoundly impacts patient prognosis. The liver is the most common site of metastatic disease across the range of solid tumors, and approximately 50% of patients with metastatic cancer harbor liver metastases. The advent of immune checkpoint inhibitors (ICIs) has considerably improved the prognosis of patients with advanced malignancies. However, the effect of metastatic sites, particularly liver metastases, on ICI efficacy remains uncertain. Recently, a meta-analysis by Tian et al. examined the impact of liver metastases on ICI efficacy across various tumor types. The findings indicated that, among all patients with cancer receiving ICI treatment, the presence of liver metastases was associated with an inferior prognosis. Notably, patients with urinary system tumors, such as renal cell carcinoma and UC, have the worst outcomes (22). Consistent with these data, a multicenter study showed that bone and liver metastases in patients with mUC treated with ICIs are associated with lower PFS, OS, and ORR (18). A comprehensive investigation of these aspects is essential to optimize the clinical application of ICIs and enhance therapeutic outcomes in a broader population of cancer patients. In the present study, we evaluated the efficacy of pembrolizumab and avelumab in mUC patients with liver metastases in comparison with historical controls.

### Cohort 1

Pembrolizumab, an IgG4-κ humanized monoclonal antibody that targets PD-1, received Food and Drug Administration (FDA) approval in May 2017 for the treatment of pretreated mUC patients based on the positive results of the Keynote-045 (KN045) phase III trial. In this study, the presence of liver metastases was a stratification factor, and approximately 34% of patients with this characteristic were enrolled in each arm of the trial. Unfortunately, outcomes focusing on this specific population are not available for KN 045, analysis of OS in key subgroups shows an HR 0.85 (0.61–1.20) (23). In the present real-world dataset, only 17% of the 1,341 patients treated with pembrolizumab had liver metastases. The total population outcomes (OS and ORR) of ARON-2 were improved indirectly compared to those of KN045, confirming the efficacy of pembrolizumab in this setting.

Patients with liver metastases had a median OS of 9.4 months compared with 20.1 months in patients without liver metastases (p <0.001). Available historical data on vinflunine in the same setting showed the poorest prognosis in these patients. In the CURVE trial, mUC patients with liver metastases had a mOS of 5.6 months *vs*. 9.4 months in patients without, and in the Spanish dataset, mOS was 6.1 months *vs*. 11.7 months, respectively (24, 25). These data confirm that the presence of liver metastases is still a negative prognostic factor in the immunotherapy era, even if outcomes in this setting of patients improved compared to historical controls with chemotherapy.

Further subgroup analysis of the present dataset confirmed the negative impact of other known prognostic factors, with significantly lower OS in patients with ECOG-PS ≥2 and those with concomitant bone metastases. In the present analysis, a BMI ≥25 kg/m² emerged as an independent favorable prognostic factor in patients with liver metastases who were treated with ICIs. While this observation should be interpreted cautiously, given the retrospective nature of the study, it is consistent with the growing body of evidence describing an “obesity paradox” in immuno-oncology (26, 27). Multiple retrospective and pooled analyses across tumor types, including melanoma, non-small-cell lung cancer, renal cell carcinoma, and UC, have reported improved survival outcomes in overweight and mildly obese patients receiving ICIs compared with normal-weight individuals (28). Several non-mutually exclusive mechanisms have been proposed from a biological perspective. Obesity is associated with chronic low-grade inflammation and altered adipokine secretion, including increased leptin and decreased adiponectin levels, which may promote T-cell activation and survival (29, 30). Preclinical models have demonstrated that leptin-driven signaling enhances T-cell exhaustion while simultaneously increasing PD-1 expression, paradoxically rendering tumors more susceptible to PD-1/PD-L1 blockade (31). In addition, obesity-related metabolic reprogramming may modulate cytokine profiles and immune cell trafficking within the tumor microenvironment, potentially amplifying the therapeutic effect of immune checkpoint blockade (32). Importantly, recent translational studies suggest that patients with higher adiposity exhibit increased engagement of the PD-1/PD-L1 axis and enhanced reinvigoration of exhausted CD8+ T cells after ICI therapy (33). These findings provide biological plausibility for our clinical observations and support the hypothesis that host metabolic status may act as a modifier of immunotherapy efficacy, even in traditionally immunosuppressive contexts, such as liver metastases (34). However, BMI is an imperfect surrogate for body composition and metabolic health. Future prospective studies incorporating direct measurements of adipokines (e.g., leptin and adiponectin), insulin resistance markers, and sarcopenia indices are warranted to validate the obesity paradox hypothesis and disentangle the relative contributions of adiposity, nutrition, and muscle mass to immunotherapy outcomes.

In our pembrolizumab-treated cohort with liver metastases, male patients showed a numerically longer OS than females, with a difference approaching statistical significance. Although this finding did not reach conventional levels of statistical significance and should be interpreted cautiously, it is consistent with emerging evidence suggesting that sex may influence the response to ICIs.

Biological differences between males and females, particularly in hormonal regulation and immune function, may contribute to the divergent immunotherapy outcomes. Estrogens and androgens differentially modulate T-cell activation, regulatory T-cell expansion, cytokine production, and immune checkpoint expression, thereby shaping the tumor immune microenvironment (35–37). In addition, sex-related differences in hepatic immune regulation may be especially relevant in the context of liver metastases, given the intrinsically tolerogenic role of the liver and its impact on systemic antitumor immunity.

Taken together, these observations suggest that sex may act as a potential modifier of immunotherapy efficacy in patients with liver metastases. However, given the limited statistical power of the subgroup analyses, our results should be regarded as hypothesis-generating. Future studies should systematically incorporate sex as a biological variable and explore the interaction effects between sex and metastatic sites in multivariable models.

### Cohort 2

Avelumab, a human IgG1 monoclonal antibody that binds PD-L1, received FDA approval in June 2020 for maintenance treatment of patients with mUC who have not progressed during first-line platinum-based chemotherapy (38). Approval was based on the positive results of the phase III JAVELIN Bladder 100 trial, which showed a significantly prolonged OS and progression-free survival (PFS) of avelumab versus Best Supportive Care alone in this patient population. There are no available data on efficacy outcomes in patients with liver metastases; in the trial, this characteristic was included in “visceral metastases” which were present in 55% of patients enrolled (39).

In ARON-2, 18% of patients had liver metastases, a slightly higher percentage compared to other real-world experiences, such as READY and AVENANCE, which included 12.8% and 15% of liver as metastatic sites, respectively. The READY trial reported only an HR of 2.85 (95%CI: 1.75–4.62) comparing OS in patients with liver metastasis versus those without, and no specific data have been reported in the AVENANCE trial (40, 41). In the avelumab-treated group in ARON-2, patients with liver metastases exhibited significantly lower OS than those without hepatic involvement (16.4 months *vs*. 27.0 months, p <0.001). However, in the subgroup of patients with liver metastases, a higher BMI and better ECOG-PS remained favorable prognostic factors.

Switch maintenance in mUC is a “new” validated treatment strategy, and there is a known positive selection of patients who benefit from it. In addition, to better understand whether immunotherapy can improve outcomes in patients with liver metastases in this setting, we may consider a few historical data. In 1999, Bajorin validated “the Bajorin prognostic factors” by analyzing five trials of the MVAC regimen, where 11.3% of patients with liver metastases were included, showing a mOS of 9.87 months *vs*. 15.45 months without (16). A Japanese group showed an mOS of 9 months *vs*. 17 months in the same population (42). ARON-2 showed that in patients with liver metastases treated with avelumab, an improvement in OS was achieved.

When interpreting the outcomes of both cohorts, it is essential to contextualize our findings with the historical chemotherapy results in patients with liver metastases. Prior studies reported markedly inferior outcomes in this subgroup, with median OS ranging between 5.6 months and 9.8 months in patients receiving vinflunine or MVAC-based regimens. In contrast, ARON-2 demonstrated a median OS of 9.4 months in post-platinum patients treated with pembrolizumab and 16.4 months in those receiving avelumab maintenance, suggesting that immune checkpoint inhibitors provide a clinically meaningful survival improvement even in this poor-prognosis population. Although liver involvement remains a strong adverse prognostic factor, these comparisons underscore that immunotherapy has shifted the therapeutic benchmark for patients with mUC with hepatic metastases. Our results complement those of Zacchi et al., who reported comparable OS between pembrolizumab and avelumab in a real-world population (20). Although our study did not aim to directly compare the two agents, the differential prognostic impact of liver metastases across cohorts highlights the importance of disease biology and treatment timing. Consistently inferior outcomes in patients with hepatic metastases, regardless of the immunotherapy used, underscore that liver involvement reflects a biologically aggressive disease subset rather than a lack of activity of ICIs.

Numerous studies have investigated the mechanisms by which liver metastases influence the effectiveness of immune checkpoint inhibitors (ICIs). Yoshida et al., suggest that hepatic microenvironment may promote immunosuppression through mechanisms involving myeloid cells and immunosuppressive factors, thereby limiting the efficacy of immunotherapy. Furthermore, the lower response observed could reflect reduced lymphocytic infiltration in liver metastases compared with other metastatic sites (43). Lee et al. demonstrated that liver metastases significantly impair tumor-specific immunity through an antigen-specific PD-1-dependent pathway. This process is associated with the coordinated activation of regulatory T-cells and alterations in intratumoral CD11b+ monocytes (44). Similarly, Tumeh et al. found that liver metastases were correlated with a reduced presence of CD8+ T-cells at the invasive margin of distant tumors (45). Furthermore, Yu et al. revealed that liver metastases recruit immunosuppressive macrophages that facilitate antigen-specific T cell apoptosis, leading to systemic depletion of T cells and diminished immunotherapy efficacy (19).

Despite the robustness of the collected data, this study has some limitations that should be considered. First, the retrospective nature of the analysis introduces potential biases, limiting the ability to establish definitive causal relationships between the variables. Additionally, variability in clinical practices across participating centers may have influenced the results, although the adoption of standardized data-collection criteria has mitigated this risk. The limited sample size of the sex-stratified subgroups, particularly among patients with liver metastases, may have reduced the statistical power to fully assess sex-specific differences in immunotherapy outcomes. Another limitation is the lack of detailed data on predictive biomarkers, such as PD-L1 expression levels and molecular characteristics, or concomitant treatments that may influence the response to immunotherapy. Finally, a limitation of the present analysis is the absence of systematic data on patients’ pre-existing hepatic conditions, such as fatty liver disease, cirrhosis, or chronic inflammatory liver disease. These conditions may influence both tumor biology and the tolerance or response to systemic therapy.

Although liver metastases remain a strong adverse prognostic factor, our findings suggest that ICIs provide clinically meaningful benefits compared to historical chemotherapy data. These results support the need for more individualized therapeutic strategies for this high-risk population.

Based on the present data, several hypotheses can be proposed. First, the integration of ICIs with loco-regional treatments for liver metastases, such as stereotactic radiotherapy or ablative techniques, may enhance systemic antitumor immunity by promoting antigen release and overcoming liver-mediated immune tolerance. Second, the combination of immunotherapeutic approaches, including dual immune checkpoint blockade or the addition of agents targeting immunosuppressive pathways (e.g., CTLA-4 or TGF-β inhibition), may represent rational strategies to improve outcomes in patients with hepatic involvement.

Prospective biomarker-driven studies are warranted to validate these approaches and refine treatment selection for patients with metastatic urothelial carcinoma and liver metastases.

## Conclusion

The results of this real-world study confirm the negative prognostic role of liver metastases in immunotherapy. Compared to historical data with chemotherapy, treatment with pembrolizumab or avelumab seems to improve outcomes in patients with mUC and liver metastases. The presence of liver metastases was associated with significantly worse OS than in patients without hepatic involvement in both analyzed cohorts. These findings reinforce the unmet need and importance of personalized therapeutic strategies for patients with hepatic metastases. Further studies are warranted to explore treatment approaches that could improve outcomes in this patient population with the goal of optimizing clinical management.

## Data Availability

The raw data supporting the conclusions of this article will be made available by the authors, without undue reservation.
