# Supplementary material for: Liver metastases in advanced urothelial carcinoma (ARON-2): do pembrolizumab and avelumab make a difference in a poor-prognosis scenario?
Source: Front Immunol. 2026 Feb 24;17:1667155. doi: 10.3389/fimmu.2026.1667155 (PMC12971714; doi:10.3389/fimmu.2026.1667155)
Supplement: Supplementary file 1 [file Table1.docx]

**Supplementary Materials**

**Table S1.** Univariable and multivariable analyses of Cohort 1 and 2.

| **Overall Survival (Overall population)** | **Univariable Cox Regression** | | **Multivariable Cox Regression** | |
| --- | --- | --- | --- | --- |
|  | **HR (95%CI)** | ***p-value*** | **HR (95%CI)** | ***p-value*** |
| Sex (females vs males) | 1.27 (0.91−1.53) | 0.141 |  |  |
| Age ≥70y (Y vs N) | 1.05 (0.89−1.29) | 0.359 |  |  |
| BMI (> 25 vs ≤ 25 kg/m^2^) | 0.65 (0.38−0.93) | **0.004** | 0.69 (0.54−0.88) | **0.001** |
| Smokers vs no-smokers | 0.89 (0.73−1.14) | 0.221 |  |  |
| ECOG PS (≥ 2 vs 0-1) | 2.95 (2.40−4.12) | **<0.001** | 2.91 (2.20−3.67) | **<0.001** |
| Histology (pure UC vs variants) | 0.91 (0.71−1.31) | 0.545 |  |  |
| Upper vs Lower urinary tract | 1.00 (0.81−1.32) | 0.759 |  |  |
| Synchronous metastatic disease (Y vs N) | 1.30 (1.01−1.59) | **0.019** | 1.20 (1.02−1.39) | **0.022** |
| Lymph node (Y vs N) | 0.86 (0.70−1.13) | 0.145 |  |  |
| Lung metastases (Y vs N) | 1.22 (1.02−1.33) | **0.031** | 1.08 (0.98−1.32) | 0.269 |
| Bone metastases (Y vs N) | 1.50 (1.19−1.99) | **<0.001** | 1.43 (1.20-1.65) | **<0.001** |
| Liver metastases (Y vs N) | 1.85 (1.41−2.90) | **< 0.001** | 1.55 (1.18-2.05) | **<0.001** |
| Brain metastases (Y vs N) | 2.82 (1.76−4.51) | **<0.001** | 2.84 (1.76-4.58) | **<0.001** |
| Type of immunotherapy (pembrolizumab vs avelumab) | 1.45 (0.98−1.97) | 0.101 |  |  |
| Propensity Score | 0.95 (0.91−1.01) | 0.122 |  |  |
| BMI = Body Mass Index; ECOG-PS = Eastern Cooperative Oncology Group-Performance Status; Y = yes; N = no | | | | |

**Table S2.** Univariable and multivariable analyses of Cohort 1.

| **Overall Survival (Overall population)** | **Univariable Cox Regression** | | **Multivariable Cox Regression** | |
| --- | --- | --- | --- | --- |
|  | **HR (95%CI)** | ***p-value*** | **HR (95%CI)** | ***p-value*** |
| Sex (females vs males) | 1.12 (0.95−1.32) | 0.173 |  |  |
| Age ≥70y (Y vs N) | 1.08 (0.93−1.26) | 0.286 |  |  |
| BMI (> 25 vs ≤ 25 kg/m^2^) | 0.80 (0.68−0.93) | **0.005** | 0.79 (0.67−0.92) | **0.002** |
| Smokers vs no-smokers | 0.88 (0.76−1.02) | 0.010 |  |  |
| ECOG PS (≥ 2 vs 0-1) | 2.71 (2.20−3.35) | **<0.001** | 2.94 (2.36−3.66) | **<0.001** |
| Histology (pure UC vs variants) | 0.91 (0.76−1.10) | 0.336 |  |  |
| Upper vs Lower urinary tract | 0.99 (0.83−1.17) | 0.869 |  |  |
| Synchronous metastatic disease (Y vs N) | 1.27 (1.09−1.48) | **0.002** | 1.25 (1.07−1.47) | **0.006** |
| Lymph node (Y vs N) | 0.86 (0.73−1.01) | 0.064 |  |  |
| Lung metastases (Y vs N) | 1.27 (1.08−1.48) | **0.003** | 1.12 (0.96−1.32) | 0.156 |
| Bone metastases (Y vs N) | 1.52 (1.30−1.78) | **<0.001** | 1.44 (1.22-1.69) | **<0.001** |
| Liver metastases (Y vs N) | 1.75 (1.46−2.09) | **< 0.001** | 1.46 (1.21-1.75) | **<0.001** |
| Brain metastases (Y vs N) | 2.82 (1.76−4.51) | **<0.001** | 2.84 (1.76-4.58) | **<0.001** |
| BMI = Body Mass Index; ECOG-PS = Eastern Cooperative Oncology Group-Performance Status; Y = yes; N = no | | | | |

**Table S3.** Univariable and multivariable analyses of advanced UC patients with liver metastases from Cohort 1.

| **Overall Survival (Overall population)** | **Univariable Cox Regression** | | **Multivariable Cox Regression** | |
| --- | --- | --- | --- | --- |
|  | **HR (95%CI)** | ***p-value*** | **HR (95%CI)** | ***p-value*** |
| Sex (females vs males) | 1.39 (0.98−1.97) | 0.066 |  |  |
| Age ≥70y (Y vs N) | 1.12 (0.81−1.54) | 0.492 |  |  |
| BMI (> 25 vs ≤ 25 kg/m^2^) | 0.69 (0.50−0.96) | **0.027** | 0.79 (0.68−0.92) | **0.003** |
| Smokers vs no-smokers | 0.90 (0.65−1.25) | 0.536 |  |  |
| ECOG PS (≥ 2 vs 0-1) | 2.83 (1.89−4.27) | **<0.001** | 2.95 (2.37−3.66) | **<0.001** |
| Histology (pure UC vs variants) | 0.98 (0.66−1.46) | 0.935 |  |  |
| Upper vs Lower urinary tract | 1.04 (0.74−1.47) | 0.816 |  |  |
| Synchronous metastatic disease (Y vs N) | 1.55 (1.12−2.13) | **0.008** | 1.31 (1.12−1.53) | **0.001** |
| Lymph node (Y vs N) | 1.00 (0.72−1.39) | 0.997 |  |  |
| Lung metastases (Y vs N) | 1.17 (0.85−1.61) | 0.322 |  |  |
| Bone metastases (Y vs N) | 1.45 (1.03−2.02) | **0.031** | 1.41 (1.20-1.67) | **<0.001** |
| Brain metastases (Y vs N) | 1.81 (0.84−3.87) | 0.129 |  |  |
| BMI = Body Mass Index; ECOG-PS = Eastern Cooperative Oncology Group-Performance Status; Y = yes; N = no | | | | |

**Table S4.** Univariable and multivariable analyses of Cohort 2.

| **Overall Survival (Overall population)** | **Univariable Cox Regression** | | **Multivariable Cox Regression** | |
| --- | --- | --- | --- | --- |
|  | **HR (95%CI)** | ***p-value*** | **HR (95%CI)** | ***p-value*** |
| Sex (females vs males) | 1.44 (0.96−2.15) | 0.081 |  |  |
| Age ≥70y (Y vs N) | 0.92 (0.64−1.32) | 0.643 |  |  |
| BMI (> 25 vs ≤ 25 kg/m^2^) | 0.28 (0.19−0.43) | **<0.001** | 0.36 (0.24−0.55) | **<0.001** |
| Smokers vs no-smokers | 0.91 (0.62−1.32) | 0.605 |  |  |
| ECOG PS (≥ 2 vs 0-1) | 3.31 (2.40−4.59) | **<0.001** | 2.65 (1.91−3.68) | **<0.001** |
| Histology (pure UC vs variants) | 0.92 (0.55−1.54) | 0.757 |  |  |
| Upper vs Lower urinary tract | 1.10 (0.75−1.62) | 0.635 |  |  |
| Synchronous metastatic disease (Y vs N) | 1.37 (0.95−1.97) | 0.092 |  |  |
| Lymph node (Y vs N) | 0.87 (0.60−1.28) | 0.487 |  |  |
| Lung metastases (Y vs N) | 1.20 (0.84−1.73) | 0.318 |  |  |
| Bone metastases (Y vs N) | 1.44 (0.96−2.15) | 0.075 |  |  |
| Liver metastases (Y vs N) | 2.07 (1.32−3.23) | **0.001** | 1.69 (1.07-2.68) | **0.025** |
| BMI = Body Mass Index; ECOG-PS = Eastern Cooperative Oncology Group-Performance Status; Y = yes; N = no | | | | |

**Table S5.** Univariable and multivariable analyses of advanced UC patients with liver metastases from Cohort 2.

| **Overall Survival (Overall population)** | **Univariable Cox Regression** | | **Multivariable Cox Regression** | |
| --- | --- | --- | --- | --- |
|  | **HR (95%CI)** | ***p-value*** | **HR (95%CI)** | ***p-value*** |
| Sex (females vs males) | 0.61 (0.23−1.62) | 0.316 |  |  |
| Age ≥70y (Y vs N) | 0.91 (0.41−2.04) | 0.826 |  |  |
| BMI (> 25 vs ≤ 25 kg/m^2^) | 0.27 (0.09−0.80) | **0.018** | 0.38 (0.13−1.13) | 0.081 |
| Smokers vs no-smokers | 2.13 (0.91−4.97) | 0.080 |  |  |
| ECOG PS (≥ 2 vs 0-1) | 3.57 (1.88−6.81) | **<0.001** | 3.11 (1.62−5.96) | **<0.001** |
| Histology (pure UC vs variants) | 0.87 (0.30−2.55) | 0.797 |  |  |
| Upper vs Lower urinary tract | 0.27 (0.06−1.15) | 0.077 |  |  |
| Synchronous metastatic disease (Y vs N) | 1.38 (0.61−3.14) | 0.443 |  |  |
| Lymph node (Y vs N) | 1.21 (0.52−2.83) | 0.626 |  |  |
| Lung metastases (Y vs N) | 1.17 (0.50−2.74) | 0.714 |  |  |
| Bone metastases (Y vs N) | 1.24 (0.52−2.99) | 0.626 |  |  |
| BMI = Body Mass Index; ECOG-PS = Eastern Cooperative Oncology Group-Performance Status; Y = yes; N = no | | | | |

**Figure S1.** Selection process from the ARON-2 dataset.

**
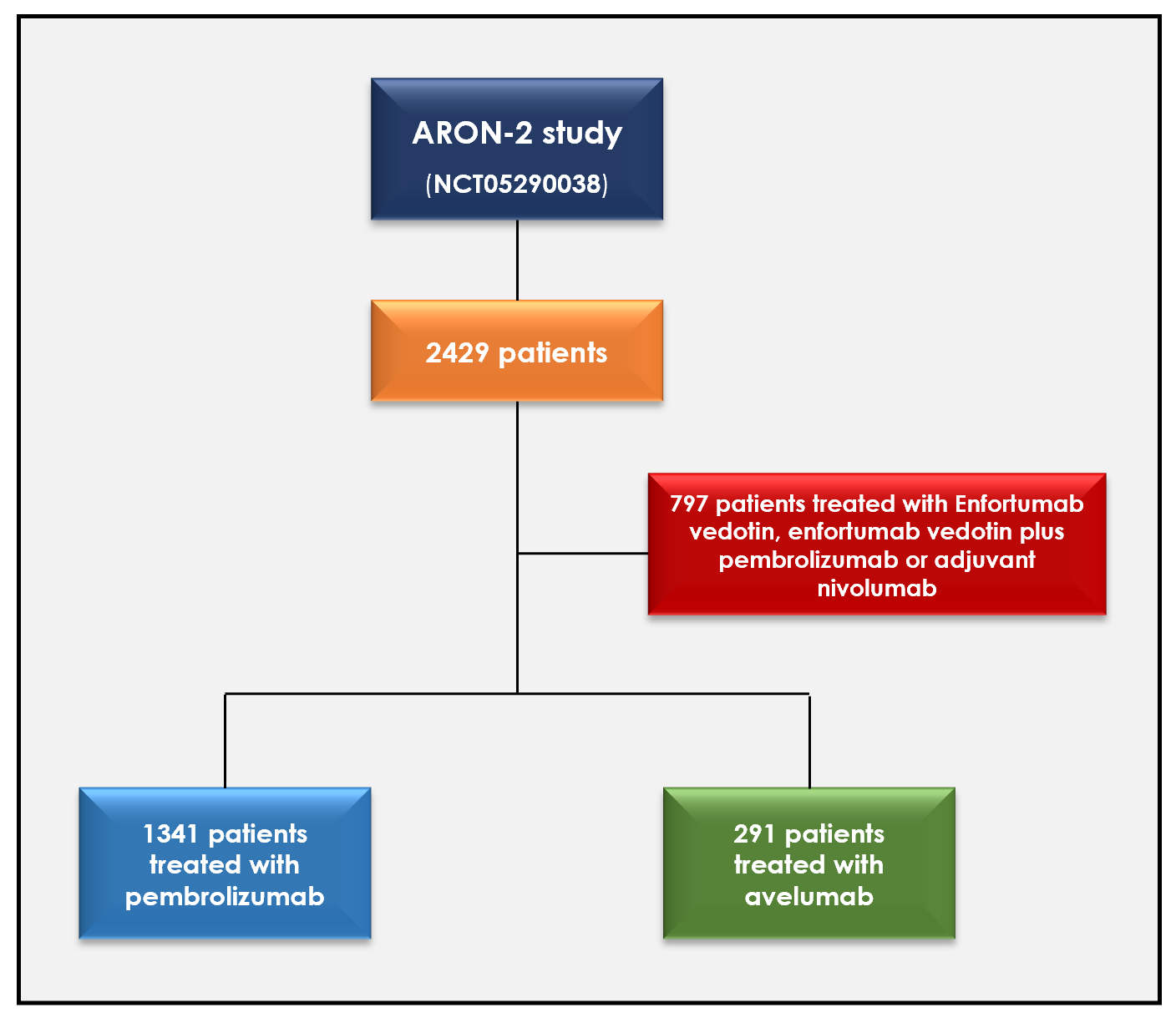
**

**Figure S2.** Map of Countries participating to the ARON-2 Study.

**
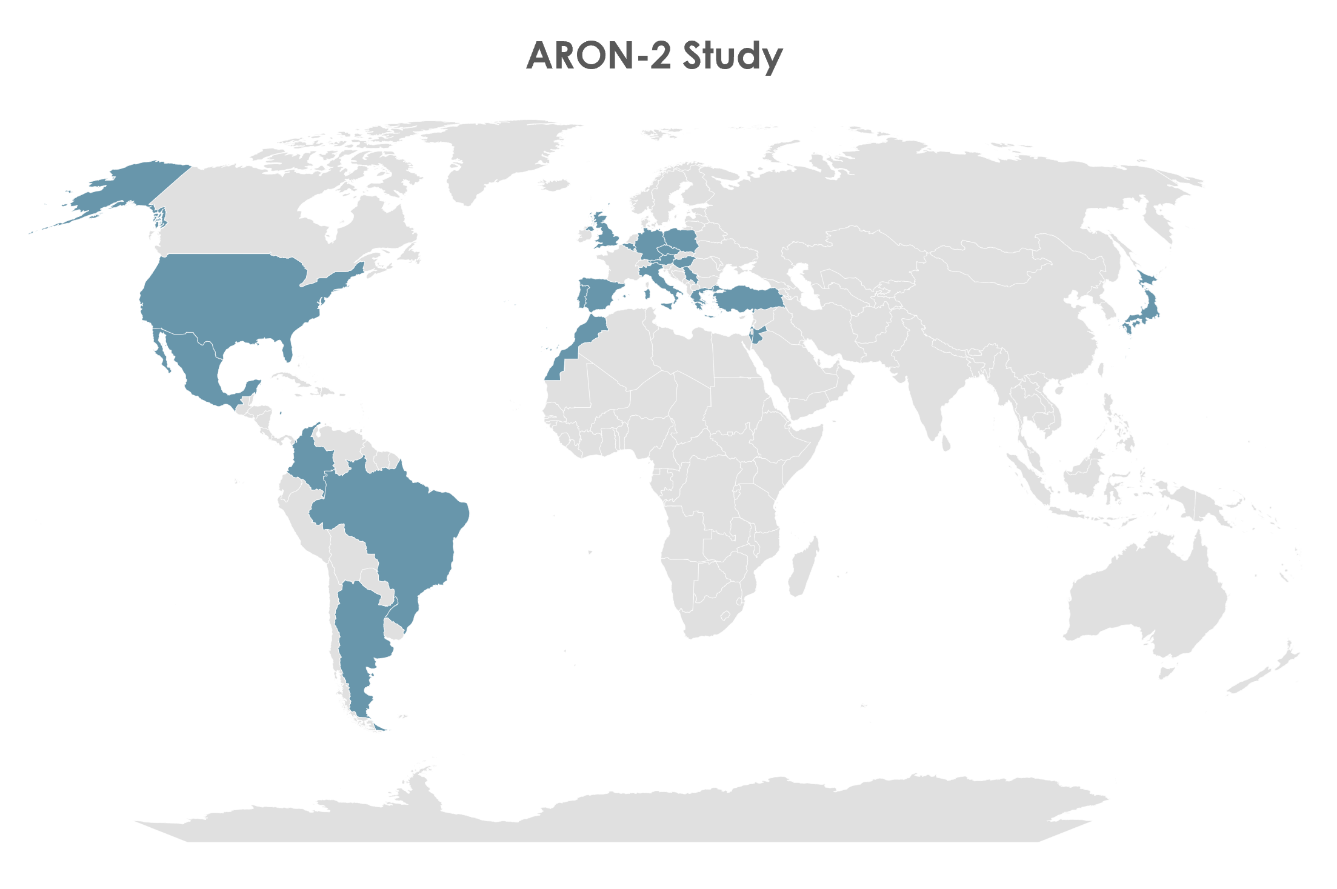
**
